# THE COVID-19 PANDEMICS’ IMPACTS ON OLDER ADULTS’ MENTAL HEALTH: THE BUFFERING EFFECTS OF NEIGHBORHOOD COHESION

**DOI:** 10.1093/geroni/igac059.2971

**Published:** 2022-12-20

**Authors:** Kedong Ding

**Affiliations:** Case Western Reserve University, Cleveland, Ohio, United States

## Abstract

**Background:**

During the COVID-19 pandemic, many older adults reported less frequency of having in-person contact due to the worries of COVID -19, which raises concerns about their mental health. The past research suggests neighborhood cohesion can facilitate social participation and have positive effects on mental health. This study aims to examine the impact of the decreased social contacts due to COVID on older adults’ depressive symptoms, and whether the relationship is moderated by neighborhood cohesion. Method: Data were from the 2020 waves of the Health and Retirement Study (HRS) (n=2,732). Participants included community-dwelling adults aged 65 years or older. The independent variable is the perceived changing amount of in-person contact due to the pandemic. Neighborhood cohesion was measured by four-item sales that assess the extent to which respondents feel about the neighborhoods and neighbors’ trust. The depressive symptoms were measured by the 8-item CES-D scale. All models were controlled for sociodemographic characteristics and health status. Multivariate regression models were used to and an interaction term was created to examine the moderating effects of neighborhood cohesion.

**Results:**

The results from the multivariate regression model show that perceived neighborhood cohesion was associated with fewer depressive symptoms (β=-.16, p < 0.001). Not having enough in-person contact since the pandemic is associated with more depressive symptoms (β=.24, p < 0.001). High perceived neighborhood cohesion attenuates the relationship between not having enough in-person contact and depressive symptoms.

**Conclusion:**

This study demonstrates the buffering effects of neighborhood cohesion on Covid-19 impacts on older adults’ in-person contact and mental health outcomes.

